# A detection method for android application security based on TF-IDF and machine learning

**DOI:** 10.1371/journal.pone.0238694

**Published:** 2020-09-11

**Authors:** Hongli Yuan, Yongchuan Tang, Wenjuan Sun, Li Liu

**Affiliations:** 1 Institute of information engineering, Anhui Xinhua University, Hefei, Anhui, China; 2 School of Big Data & Software Engineering, Chongqing University, Chongqing, China; 3 Department of Modern Mechanics, University of Science and Technology of China, Hefei, Anhui, China; Xidian University, CHINA

## Abstract

Android is the most widely used mobile operating system (OS). A large number of third-party Android application (app) markets have emerged. The absence of third-party market regulation has prompted research institutions to propose different malware detection techniques. However, due to improvements of malware itself and Android system, it is difficult to design a detection method that can efficiently and effectively detect malicious apps for a long time. Meanwhile, adopting more features will increase the complexity of the model and the computational cost of the system. Permissions play a vital role in the security of the Android apps. Term Frequency—Inverse Document Frequency (TF-IDF) is used to assess the importance of a word for a file set in a corpus. The static analysis method does not need to run the app. It can efficiently and accurately extract the permissions from an app. Based on this cognition and perspective, in this paper, a new static detection method based on TF-IDF and Machine Learning is proposed. The system permissions are extracted in Android application package’s (Apk’s) manifest file. TF-IDF algorithm is used to calculate the permission value (PV) of each permission and the sensitivity value of apk (SVOA) of each app. The SVOA and the number of the used permissions are learned and tested by machine learning. 6070 benign apps and 9419 malware are used to evaluate the proposed approach. The experiment results show that only use dangerous permissions or the number of used permissions can’t accurately distinguish whether an app is malicious or benign. For malware detection, the proposed approach achieve up to 99.5% accuracy and the learning and training time only needs 0.05s. For malware families detection, the accuracy is 99.6%. The results indicate that the method for unknown/new sample’s detection accuracy is 92.71%. Compared against other state-of-the-art approaches, the proposed approach is more effective by detecting malware and malware families.

## 1 Introduction

The number of smartphone users is growing rapidly. It is expected to grow from 2.7 billion in 2016 to 6 billion in 2020 [1]. The open-source mobile operating system Android is very popular among mobile users and developers. In 2017, 85.0% of new smartphones sold used Android operating system [2]. Continuous increase in the number of Android apps. Android users are able to choose between 3.8 million apps at the first quarter of 2018 [3]. Meanwhile, due to the openness of the Android system, the download of third-party Android apps has increased dramatically in recent years. Unfortunately, the third-party Android apps markets are poorly regulated. Lack of the supervision leads to the increasingly security issues of Android apps. Malware authors use stealth techniques, dynamic execution, etc. to bypass the existing protection methods [4]. Malware can cause Android users’ privacy disclosure, short message service (SMS) interception (which can lead to account theft, network payment security issues), malicious deduction, automatic transmission of virus links to other people in the address book, etc. Therefore, a fast and efficient detection system is extremely needed [5].

Android permissions play an important role in protecting the security of Android apps [6, 7]. Research shows that more than >70% of Android apps request permissions they don’t need [1]. Currently, when installing, Android apps tell users what permissions will be use. However, these still can’t guarantee the security of Android apps [8].

There are some researches based on permissions for above issues. W. Enck et al. [9] proposed an Android security framework based on apps’ permissions (called Kirin system). Kirin system can detect malicious apps and automatically checks dangerous permissions when installed. However, the permission check lead to the user install the apps need more time. Y. Zhang et al. [10, 11] proposed the VetDroid to reconstruct the sensitive behavior of Android apps. However, the dynamic analysis platform requires all execution paths. R. Zhang et al. [12] proposed a scheme for rapid detection of malware based on the perspective of permissions correlation. Experiments on 2000 samples show that the detection accuracy is 88.98%. Tuncay G. S. et al. [6] and J. Sellwood et al. [13] studied custom permissions and solved the problem of custom permissions. Google also worked on custom permissions by issuing bug fixes. But custom permissions are only for one or several apps. Therefore Custom permissions have no commonality in detecting large-scale Android apps.

The static detection method [14] is simple and efficient compared with the dynamic and hybrid methods. Android apps permissions are declared in the manifest.xml file. The elements of the manifest.xml file can be read without running the app. So the permissions in an Android app can be quickly and accurately extracted by the static method.

In this paper, we present a new approach, that extracts permissions in apps, calculates the sensitive value of permissions (SVOA) with TF-IDF algorithm, and uses machine learning to detect malware. In the proposed approach, firstly, the Apks are decompiled by Apktool. The system permissions declared in the Manifest files are extracted by a Python program. Secondly, a model based on TF-IDF algorithm to detect the security of Android app is proposed. The permission’s sensitivity value of Apk (SVOA) is calculated by the model. Thirdly, 15489 samples (6070 benign samples and 9419 malware samples) are trained and tested by machine learning classification algorithms (include Naive Bayes (NB), J48, Bayesian Networks (BN), Random Forest (RF), Random Tree (RT) and K-Nearest Neighbor (K-NN)). Finally, the optimal algorithm is selected for the detection and analysis of malware.

In summary, the contributions of our work are as follows.

1. Based on TF-IDF, a new detection approach for malicious apps is proposed. The approach can obtain the sensitive value of each apk by calculating the SVOA in the apk. The approach could effective detected malware.
2. 15489 samples are trained and classified by six classifiers and two validation methods of machine learning, and found the optimal classifier and validation method.
3. The experimental results are analyzed and discussed in detail. The results show that the accuracy of the proposed approach for malware detection can reach 99.5%. The test and classification time reached 0.05s. The accuracy of the malware families detection is 99.6%, and the partial malware families detection accuracy is higher than other state-of-the-art approaches. The proposed approach also has good detection performance for large-scale samples and unknown/new samples.

The rest section of this paper as follows. In section 2, the related work on Android apps detection is summarized. Section 3 briefly introduced the preliminaries on Android permission and TF-IDF algorithm. In section 4, a new Android app security detection approach based on the IF-IDF is proposed. In section 5, the proposed Android app security detection method is used for machine learning classify. And subsequent works are given in section5. The conclusions are given in Section 6.

## 2 Related work

Currently, many android malware detection technologies have been proposed by the researchers. There are mainly static detection method, dynamic detection method and hybrid method.

### 2.1 Static detection

Static detection method is based on decompilation technology and doesn’t need run the apps. It analyse the code, rule matching and other operations (such as permissions, data flow, control flow, etc.). [4, 14–16].

MaMaDroid [17] used Markov chains to build API sequence model. The method learn and test through the feature obtained by API sequence model. The F-measure of MaMaDroid can reached 99%. DroidSieve [18] proposed high-quality features for malware detection and malware family detection. These features include Intents, permissions, mate-information, etc. MUDFLOW [19] used sensitive sources (include the Intents, Sinks, API, etc.) to detect new malware and its accuracy can reach 86.4%.

These static detection methods used multiple features to detect malware. Its have less cost and don’t need run apps for detect malware [7, 20].

### 2.2 Dynamic detection

In contrast to static detection, dynamic detection detects apps’ behavior at runtime. It captures and analyzes sensitive behavior in real time. Dynamic detection needs to be run in a specially built environment [10, 21, 22].

DroidCat [23] used dynamic features to detect resource obfuscation, system-call obfuscation and other obfuscation. The F1 of DroidCat can reached 97%. DroidScribe [24] analyzed the running behavior of apps by dynamic detection method and divided malware into different families.

Although dynamic detections are effective in identifying malicious behaviors, they require a lot of costs [25]. Meanwhile, for conditionally triggered malicious applications, dynamic detections are also helpless.

### 2.3 Hybrid detection

Recently, the detection based on machine learning technology has received extensive attention [26–29]. However, machine learning has high requirements for sample features. Now, more and more scholars use hybrid methods to detect Android malware [25, 30–32]. Y. Du et al. [25] first analyzed the community structures of function call graphs, then used machine learning algorithms to evaluate the performance. W. Wang et al. [32] proposed DroidEnsemble which used structural and string features for static analysis of Android apps. They extracted seven string features and used three machine learning algorithms to evaluate the performance. Afonso et al. [33] and Dash et al. [34] detected malware by dynamically obtained features. Meanwhile, some state-of-the-art approaches used dynamic and static methods to obtain features to detect malware [35, 36]. The drawbacks of the hybrid approach is that it requires additional OS system consumption and a lot of time.

More and more mining techniques and machine learning techniques are applied to detect malware [37, 38]. G. Suareztangil et al. [39] proposed a text mining method that can automatically classify malicious samples and malicious families by the code structure of apps. B. Sanz et al. [40] used text mining approach in the disassembled Android apps smali, it obtained a good result of accuracy (83.51%). W. Wang et al. [41] detected malware from three aspects by data mining. They used three ranks analyze individual permissions and collaborative permissions. J. Li et al. [27] proposed the SIGPID (Significant Permission Identification). They found that only 22 permissions are significant and used machine-learning to classify different families of malware. The precision and accuracy of the method can reach above 90%. All these studies applied mining techniques analysis code structures, smalis and permissions to detect the security of apps.

To sum up, in order to simplify the detection model and improve the detection efficiency, the approach in this paper only uses the permission feature to detect malware. Android apps’ permissions are declared in the manifest.xml file. Permissions are extracted from the manifest.xml file doesn’t need to run the app, it just needs to decompile the app. Meanwhile, the Android permissions are very suitable for static extraction. Therefore, the proposed approach uses the static method (TF-IDF algorithm) to calculate the sensitivity value of the apks. Then, Android apps are learned and classified by machine learning algorithms.

## 3 Preliminaries

In this section, the background of Android permission and TF-IDF Algorithm are introduced.

### 3.1 Android Permission

The permission declaration mechanism is an important Android security mechanism. It means that if an app wants to access the system resources, it must declare the permissions in the manifest.xml. Android system permissions are divided into three categories: normal, dangerous, signature (As shown in Table 1) [42]. Normal permissions don’t involve user privacy, they also don’t need require user authorization, such as mobile phone vibration, access to the network, etc. Whereas, dangerous permissions are involve user privacy and requires user authorization, such as reading sdcard, accessing address book, etc. Signature permissions protect the apps’s private resources. These permissions can only be granted if the requesters sign with matching certificates. Except system permissions, Android apps also adopts some custom permissions. These permissions allow apps to access some particular apps [6].

**Table 1:** Android system permission categories.

Type	Interpretation	Examples
normal	very little risk	INTERNET,BLUETOOTH, etc.
dangerous	high risk	CALL_PHONE, CAMERA, etc.
signature	Need digital signature	PLATFORM, SHARE, etc.

Before Android 6.0, when installing an Apk, the android operating system will prompt the user that the permissions of the Apk will need to install and display it in a list. However, these prompts are only prompted during installation, very few users will abandon the installation of an Apk if they see the Apk needs some sensitive permissions. Beginning in Android 6.0, users will have permission authorization when running apps, which replaces the authorization during the installation [43]. However, runtime authorization is organized in groups, not individual permission. For instance, the app has been authorized for READ_CONTACTS. When the app requests WRITE_CONTACTS, the system will directly authorize WRITE_CONTACTS, because READ_CONTACTS and WRITE_CONTACTS are in same permission group.

Unfortunately, various studies have shown that most Android apps have security issues with permissions [44–47]. Although Google offers dangerous permissions, J. Li et al. [27] study shows that normal permissions aren’t all safe, and dangerous permissions aren’t all unsafe once they appear. W. Wang et al. [41] list 22 important permissions. Among them, only 8 permissions are consistent with the 24 dangerous permissions proposed by Google. Meanwhile, the detection method is extremely difficult. Statisticed by statista: until January 2018, 43.4% of users still use the Android operating system version lower than Android 6.x.

**Fig 1:**
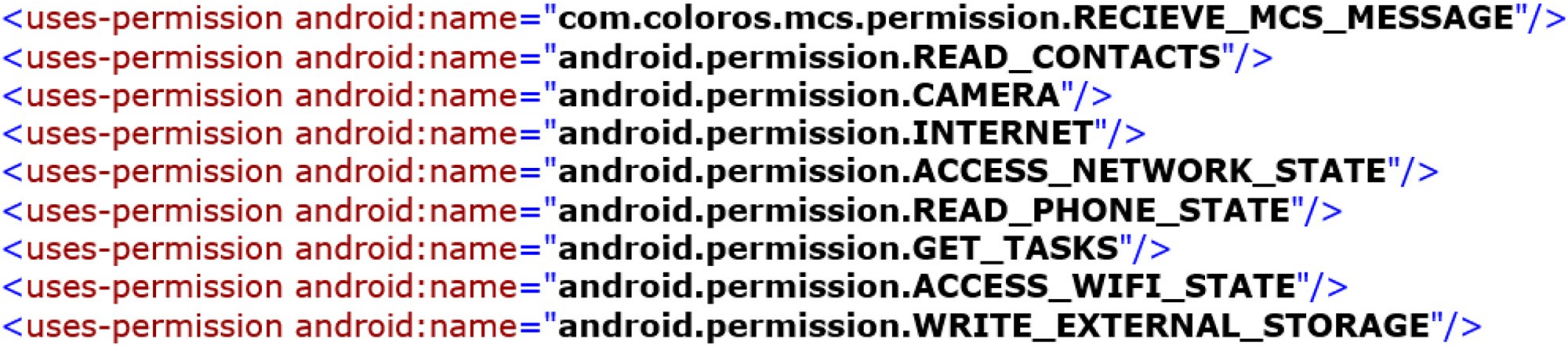
Part of the permissions declared in the Taobao app’s Manifest.xml file.

Part of the permissions which declared in the Taobao app’s Manifest.xml file are shown in Fig 1. READ_CONTACTS is reads the address book, CAMERA is takes the photo and video, and INTERNETACCESS_ NETWORK_STATE is the full network access right. Google has defined a total of 135 system permissions [48]. com.android. launcher.permission. INSTALL_SHORTCUT is a custom permission. A custom permission is used in a small range, and individual analyses are not representative [42]. Our work analyzes android system permissions, regardless of custom permissions.

### 3.2 TF-IDF algorithm

TF-IDF is a statistical method and used to evaluate the importance of a word to a document or a corpus [49, 50]. It is a commonly used weighting technique. If the number of a word occurrences in the file increases, the importance of the word increases [51].

In documents, the term frequency (TF) refers to the number of times a word appears in a document. Inverse document frequency (IDF) is a measure of the general importance of a word. A word’s IF-IDF is the value obtained by multiplying TF and IDF. The larger the IF-IDF of a word, the more important the word is in the document.

Some definitions of TF-IDF algorithm are as follows.

Let *M* be a set of documents, denoted as:

$$\begin{array}{c} M=\{d_{1},d_{2},\ldots ,d_{j},\ldots d_{\left|M\right|}\}, \end{array}$$ (1)

where *d*_*j*_ represents the *jth* document in *M*.

Let *K* be a set of terms in *M*, denoted as:

$$\begin{array}{c} K=\{t_{1},t_{2},\ldots ,t_{j},\ldots t_{\left|K\right|}\}, \end{array}$$ (2)

where *t*_*i*_ represents the *ith* in *K*.

**Definition 1**: (TermFrequency: *TF_ij_*). *TF_ij_* is frequency of *t*_*i*_ appear in *d*_*j*_, *TF_ij_* calculates formula [39, 40, 52] as follows:

$$\begin{array}{c} TF_{{}_{ij}}=\frac{n_{{}_{i,j}}}{\sum_{k}n_{{}_{k,j}}}, \end{array}$$ (3)

where *n_i,j_* is the nunber of times term *t*_*i*_ appears in a document *d*_*j*_. $\sum_{k}n_{{}_{k,j}}$ is total number of times that all terms *K* appear in the document *d*_*j*_.

**Definition 2**: (*InverseDocumentFrequency*: *IDF*_*i*_). The IDF [39, 40, 52] is the total number of documents divided by the number of documents contain the *t*_*i*_. And then take the logarithm of the quotient. The formula is as follows:

$$\begin{array}{c} IDF_{i}=\log\frac{\left|M\right|}{|j:t_{i}\in d_{j}|}, \end{array}$$ (4)

where |*M*| is the total number of documents. |*j*: *t*_*i*_ ∈ *d*_*j*_| is the number of documents contain the *t*_*i*_.

The fewer documents containing *t*_*i*_, the greater *IDF*_*i*_ value, indicating that *t*_*i*_ has a strong ability to distinguish in documents.

**Definition 3**: (*Term* Frequency—Inverse *DocumentFrequency*: *TF*−*IDF*) The *TF*−*IDF* is obtained by multiplying *TF_ij_* and *IDF*_*i*_ [39, 40, 52]. It is defined as follows:

$$\begin{array}{c} TF-IDF=TF_{{}_{ij}}\times IDF_{i}. \end{array}$$ (5)

TF-IDF combines the advantages of both TF and IDF, and evaluates the importance of *t*_*i*_ to one of the document in the documents set. The importance of *t*_*i*_ is proportional to the number of times it appears in the document and is inversely proportional to the frequency it appears in the documents set.

## 4 System model

**Fig 2:**
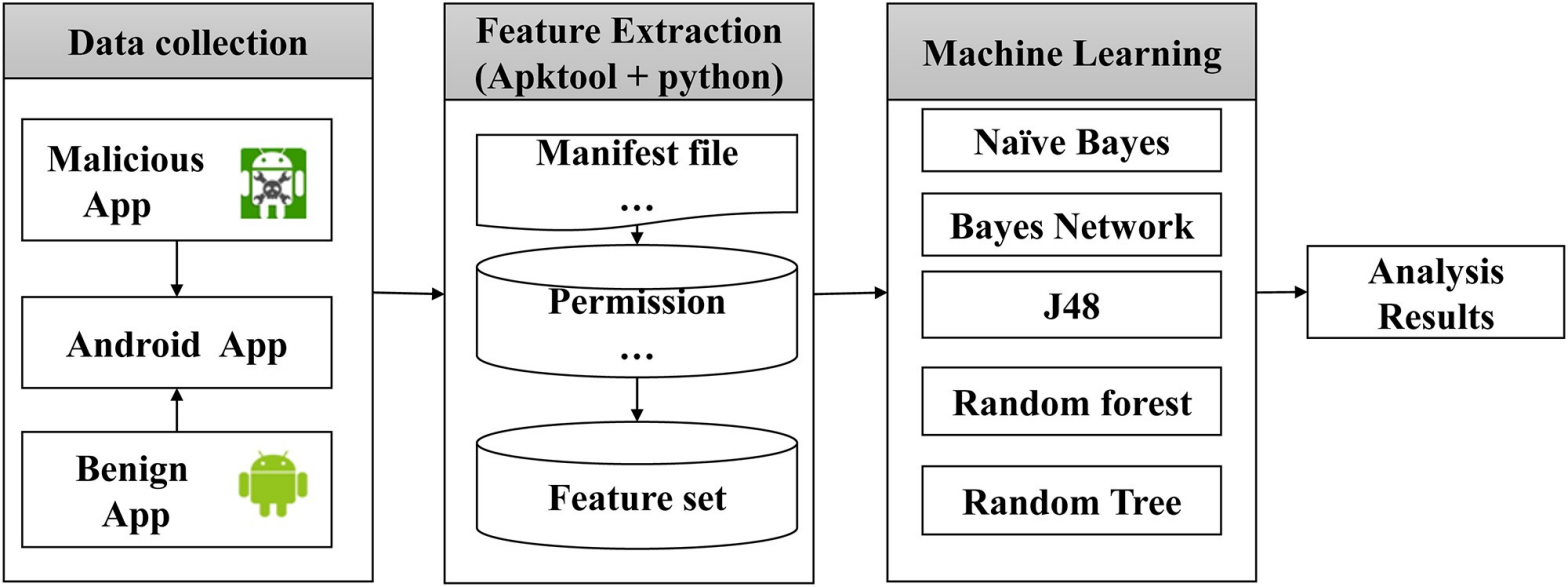
The model of the proposed system.

The proposed approach use TF-IDF to get app permission’s SVOA. The system model consists of four steps: the dataset collection, decompile Apk and calculate the Apk’s SVOA, use machine learning to test and result analysis. The system model is illustrated in Fig 2.

**Step 1. Collect Apk samples**.

This step need to write a web crawler in python and get benign Android Apks from the Android app markets (Google Play, Android third-party app stores, etc.). Then collection malicious Android Apks through university labs, research institutions and security companies.

**Step 2. Apk decompile and calculate the Apk’s SVOA**.

**Fig 3:**
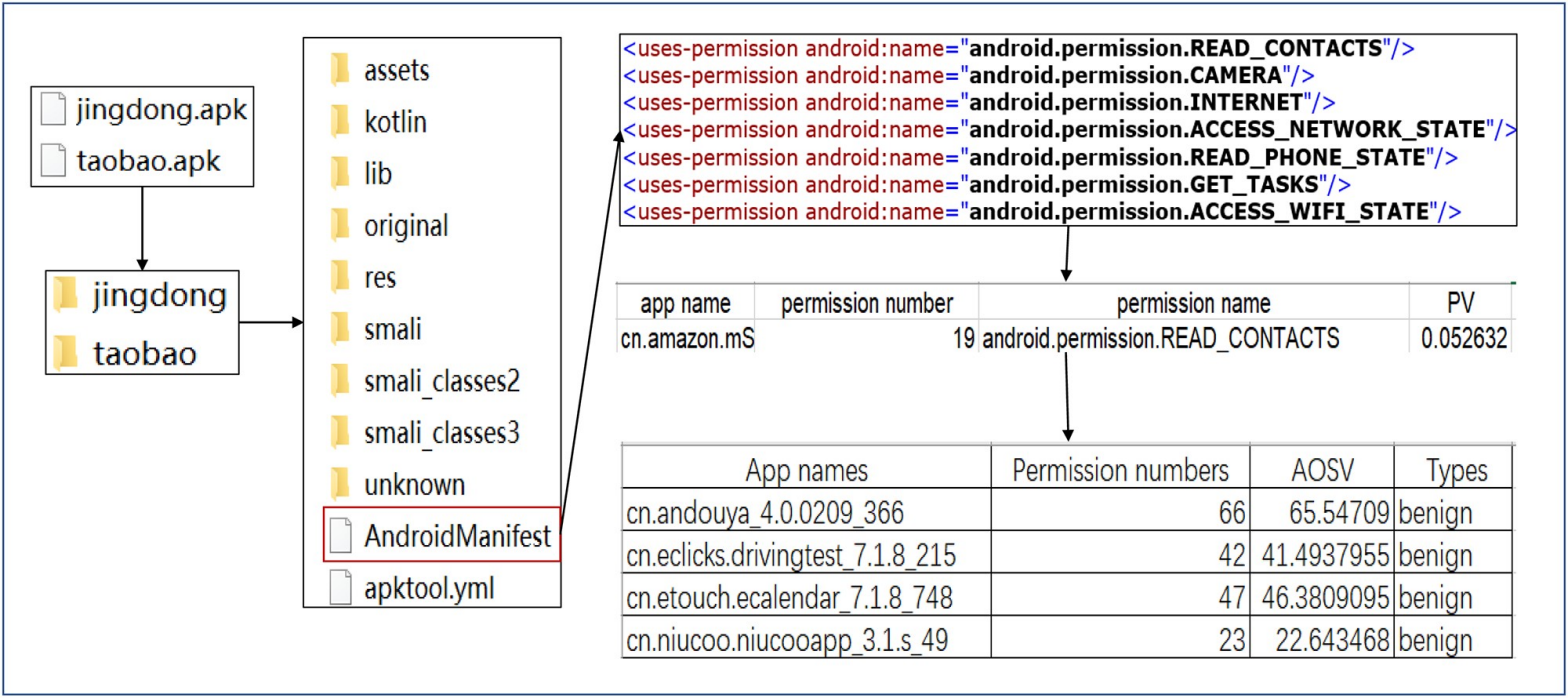
The process of calculating permission’s SVOA.

The processing flows of Apk decompile and calculate the Apk’s SVOA are shown as Fig 3. ApkTool (used to disassemble and assemble Android package (.apk)) [2] is used to decompile all the Apks in batches, and finally it generate a folder containing the manifest file, smali file, etc.

After studying Android security mechanism strategy, the declaration permissions are extracted from the Android Manifest.xml file by the Python program. Then, some custom permissions are removed and Android system permissions are retained.

The SVOA is calculated by the TF-IDF algorithm and saved as a feature set. The relevant definitions are shown as follows:

Let *W* be a set of Apks, denoted as:

$$\begin{array}{c} W=\{a_{1},a_{2},\ldots ,a_{r},\ldots ,a_{\left|W\right|}\}, \end{array}$$ (6)

where *a*_*r*_ represents the *rth* Apk in *W*. Let *U* be a set of permissions in *W*, denoted as:

$$\begin{array}{c} U=\{p_{1},p_{2},\ldots ,p_{s},\ldots ,P_{\left|U\right|}\}, \end{array}$$ (7)

where *p*_*s*_ represents the *sth* permission in *U*.

Let *V* represent the number of permissions that appear in Apk, denoted as:

$$\begin{array}{c} V=\{|V_{1}|,|V_{2}|,\ldots ,|V_{r}|,\ldots ,|V_{\left|W\right|}|\}, \end{array}$$ (8)

where |*V*_*r*_| represents the number of permissions appear *a*_*r*_.

**Definition 4**: **(Permission Frequency: PF)** The frequency of a permission (*p*_*s*_) in the Apk (*a*_*r*_) is given by

$$\begin{array}{c} PF_{sr}=\frac{\sum_{\left(p_{s}\in a_{r}\right)}\;freq\left(p_{s},a_{r}\right)}{\sum_{\left(a_{r}\in w\right)}\;freq\left(p_{s},w\right)}, \end{array}$$ (9)

where ∑_(*p*_*s*_∈*a*_*r*_)_ *freq*(*p*_*s*_, *a*_*r*_)) is the number of the permission (*p*_*s*_) occurrences in (*a*_*r*_). ∑_(*a*_*r*_∈*w*)_ *freq*(*p*_*s*_, *w*) is total number of a permission (*p*_*s*_) in *W*.

**Definition 5**: **(Permission Frequency of all: PFOA)**, PFOA denoted as:

$$\begin{array}{c} PFOA_{s}=\log\frac{\left|W\right|}{|s:p_{s}\in a_{r}|}, \end{array}$$ (10)

where |*W*| is total number of Apks *W*. |*s*: *p*_*s*_ ∈ *a*_*r*_| is number of the Apks with permission *p*_*s*_ in it.

**Definition 6**:**(Permission’s Value: PV)**, Value of permission (*p*_*s*_) in the Apk (*a*_*r*_), denoted as:

$$\begin{array}{c} PV_{s}=PF_{\left(sr\right)}\times \;PFOA_{s}, \end{array}$$ (11)

the larger the value of *PV*_*s*_, the more important *p*_*s*_ is in *a*_*r*_. This makes it easier to distinguish *a*_*r*_ from other Apks.

**Definition 7**:**(Sensitivity Value of Apk: SVOA)**, Sensitivity value of the Apk (*a*_*r*_).

$$\begin{array}{c} SVOA_{r}=\sum_{s=1}^{|V_{r}|}PV_{s}, \end{array}$$ (12)

where |*V*_*r*_| is the total number of the permissions in *a*_*r*_.

**Step 3. Machine learning classification**.

The dataset are trained and tested by Naive Bayes (NB), Bayesian Network (BN), J48, Random Tree (RT), Random Forest (RF) and *K*-Nearest Neighbor (*K*-NN) machine learning classification algorithms. The test method uses Percentage Split test method and *K*-fold cross-validation test method.

**Step 4. Experimental results evaluation and analysis**.

Using the True Positives Rate, False Positives Rate, Precision, F-Measure, Accuracy, Recall and AUC indicators to evaluate the experimental results of each machine learning. Selection an optimal model to detect and analyze malware.

## 5 Evaluation and discussion

This section mainly includes the datasets, experimental methods and parameters, evaluation system, experimental results and discussion.

### 5.1 Datasets

In this section, the datasets used in the experiment are introduced, which includes 15489 Apks (6070 benign samples and 9419 malware samples). The datasets collected from October 2012 to June 2018.

Benign samples includes 6070 Apks. They are collected in Yingyongbao [53] and Wandoujia [54]. There are some errors during the download and decompilation. We got 6,070 usable Apks out of 6,500. We used malware detection tools (VirusTotal [55] and Virscan [56]) to scan and confirm that these Apks are benign samples.

Malicious Apks are collected from Drebin dataset [57] and Virus share [58] from October 2010—August 2018. There are some errors during the download and decompilation. We got 9419 usable Apks out of 10897.

The information of the datasets is shown in Table 2. We divided the dataset into 5 datasets for different experiments. In addition to Dataset4, the other 4 datasets consist of benign apps and malicious apps. Dataset1 is used to select the optimal classifier and validation method. Dataset2 and Dataset3 combined with Dataset1 are used to verify that the proposed approach has good detection accuracy for different datasets sizes. Dataset4 is used to verify the detection of malware families by the proposed approach. Dataset5 is used to verify the detection performance of the proposed approach for currently unknown and new apps.

**Table 2:** Information of datasets.

Name	Source of Ben./Mal.	Number of Ben./Mal.	Total number
Dataset1	YingYongBao/Denbin-5	589/556	1145
Dataset2	YingYongBao/Denbin-0,1	2010/1984	3994
Dataset3	YingYongBao/Denbin-0, 1, 2, 3,4,5	5601/4530	10131
Dataset4	-/Denbin-malware_families	-/4466	4466
Dataset5	WanDouJia/Virus Share	469/423	892

### 5.2 Experimental methods and parameter design

The parameters which are used by the classifiers are shown in Table 3.

**Table 3:** 5 classifier parameter settings.

Algorithm	Parameter
Naive Bayes (NB)	*N*/*A*
Bayesian Network (BN)	*K*2
Random Tree (RT)	*m* = *log*_2_(*predictors*)+1
J48	*C*4.5
Random Forest (RF)	*N* = 100
*K* -Nearest Neighbor (*K* -NN)	*K* = 9

The experimental method of this paper uses Percentage Slit and *K*-fold cross validation. Percentage Split divides the experimental dataset into two parts according to a certain percentage, one for the training set and the other for the test set. *K*-fold cross validation randomly divides experimental samples into *K* disjoint subsets, The *K*-1 subsets are used for training and one subset is used for testing. The parameters used in the experimental method are shown in Table 4.

**Table 4:** Experimental validation methods and parameters.

Name	Parameter
*K* -fold cross validation	*K* = 10
Percentage Split	Percentage split = 66%

### 5.3 Model evaluation metrics

Following evaluation metrics are selected to evaluate the proposed approach. The classification algorithm evaluation terms are shown in Table 5. Here, the benign sample is positive and the malicious sample is negative. Using “Actual” to indicate the actual apps situation and “Predicted” to indicate the predicted apps situation. Other evaluation indicators are defined as follows.

**Table 5:** The classification algorithm evaluation terms.

	Positive (Predicted)	Negative (Predicted)
Positive (Actual)	True Positive (*TP*)	False Negative (*FN*)
Negative (Actual)	False Positive (*FP*)	True Negative (*TN*)

**Definition 8**: **(TPR)** The TP Rate represents the true positive rate that is correctly classified by the classifier. which is:

$$\begin{array}{c} TPR=\frac{TP}{TP+FN} \end{array}$$ (13)

**Definition 9**: **(FPR)** FP Rate represents the false positives rate that is incorrectly identified as Positive. which is:

$$\begin{array}{c} FPR=\frac{FP}{FP+TN} \end{array}$$ (14)

**Definition 10**: **(Precision)** Precision is the number of positive samples detected that are positive. In general, the higher the Precision, the better the classifier will work. which is:

$$\begin{array}{c} Precision=\frac{TP}{TP+FP} \end{array}$$ (15)

**Definition 11**: **(Recall)** Recall is a measure of completeness, indicating the percentage of positive tuples marked as positive. which is

$$\begin{array}{c} Recall=\frac{TP}{TP+FN} \end{array}$$ (16)

**Definition 12**: **(F-Measure)** F-measure is the weighted harmonic mean of Precision and Recall which is

$$\begin{array}{c} F-Measure=\frac{2\times Precision\times Recall}{Precision+Recall} \end{array}$$ (17)

**Definition 13**: **(Accuracy)** Accuracy is the percentage of tuples that are correctly classified by the classifier. which is:

$$\begin{array}{c} Accuracy=\frac{TP+TN}{TP+FP+TN+FN} \end{array}$$ (18)

**Definition 14**: **(Area Under Curve (AUC))** is defined as the Area Under ROC Curve. The ROC curve does not clearly indicate which classifiers perform better. But AUC can better evaluate the classifier. The greater the AUC, the better the classifier.

In fact, *Recall* = *TPR*, which is currently assigned to the positive sample category, the true positive sample as a percentage of all positive samples, also called the recall rate (how many positive sample ratios are recalled). Accuracy is the percentage of all samples that are correctly predicted for the correct sample, and represents the differentiating ability of a classifier (where the differentiating ability is not biased to positive or negative examples). Precision—recall is actually two evaluation indicators, but they are generally used simultaneously. Ideally, both are high, but generally high accuracy and low recall, or low recall and high accuracy. In cases where both requirements are high, it can be measured in terms of *F*−*Measure*.

### 5.4 Extraction and analysis of permissions

We extracted and analyzed permissions in the dataset1. The top 20 most-used system permissions statistics are shown in Table 6.

**Table 6:** The top 20 most-used system permissions in benign apps and malicious apps.

Benign	Malicious
1.ACCESS_NETWORK_STATE	1.INTERNET
2.INTERNET	2.READ_PHONE_STATE
3.WRITE_EXTERNAL_STORAGE	3.WRITE_EXTERNAL_STORAGE
4.READ_PHONE_STATE	4.ACCESS_NETWORK_STATE
5.ACCESS_WIFI_STATE	5.SEND_SMS
6.WAKE_LOCK	6.RECEIVE_BOOT_COMPLETED
7.VIBRATE	7.ACCESS_WIFI_STATE
8.GET_TASKS	8.RECEIVE_SMS
9.ACCESS_COARSE_LOCATION	9.WAKE_LOCK
10.WRITE_SETTINGS	10.READ_SMS
11.READ_EXTERNAL_STORAGE	11.ACCESS_COARSE_LOCATION
12.CHANGE_WIFI_STATE	12.ACCESS_FINE_LOCATION
13.ACCESS_FINE_LOCATION	13.VIBRATE
14.CAMERA	14.WRITE_SMS
15.MOUNT_UNMOUNT_FILESYSTEMS	15.READ_CONTACTS
16.SYSTEM_ALERT_WINDOW	16.CHANGE_WIFI_STATE
17.RECEIVE_BOOT_COMPLETED	17.INSTALL_PACKAGES
18.READ_LOGS	18.RESTART_PACKAGES
19.RECORD_AUDIO	19.GET_TASKS
20.CHANGE_NETWORK_STATE	20.CALL_PHONE

It can be seen from the Table 6, more dangerous permissions types are used in malicious apps than benign apps, such as SEND_SMS, RECEIVE_SMS, READ_SMS, CALL_PHONE, etc. However, some normal permissions have high usage rates in both benign and malicious apps, such as ACCESS_ NETWORK_STATE, READ_PHONE_STATE, VIBRATE, ACCESS_WIFI_STATE, INTERNET, GET_TASKS, WRITE_EXTERNAL_ STORAGE, etc. Therefore, it is not feasible to distinguish between malicious and benign apps based solely on the used number of dangerous permissions.

**Fig 4:**
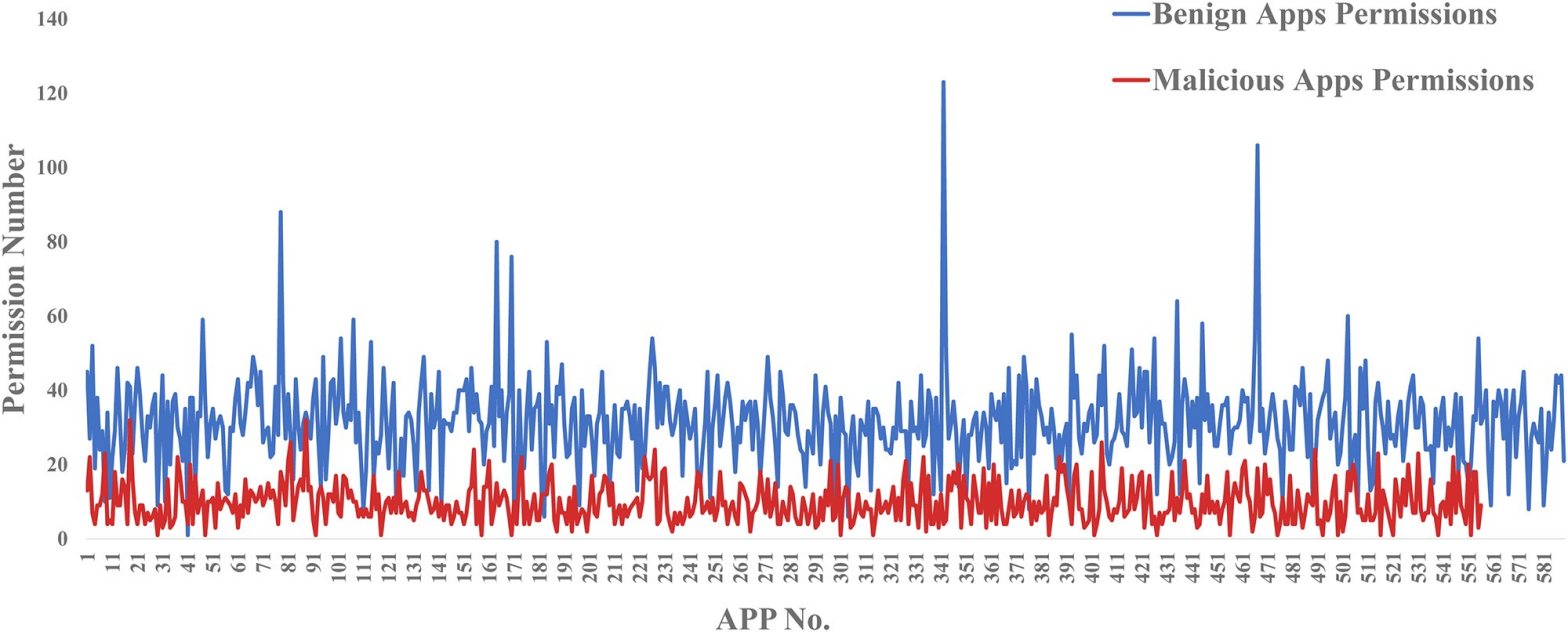
Benign and malicious apps’ permissions number.

The number of system permissions used in each benign and malicious apps (dataset1) is shown in Fig 4.

It can be obtained from Fig 4, the number of apps’ permissions for in dataset1 is less than 135 (all system permissions declarated by google). Fig 4 shows that the malicious apps use fewer permissions than the benign apps. The benign apps use an average of 35 system permissions per app in the dataset1. However, malicious apps use an average of 10 system permissions per app. This is different from K. Tam et al. [20] study: After the statistical analysis of million apps from 2010 to 2014, it is found that malicious apps use 12.99 permissions on average, and benign apps use 4.5 permissions on average. There are several reasons for this phenomenon. Firstly, different samples are used. Secondly, as time goes by, the functions of apps become more and more comprehensive, so more permissions are invoked. Accordingly, it is also not feasible to distinguish between malicious or benign apps based solely on the number of permissions used.

There exist many approaches for detecting Android malapps by extracting permissions. Some researchers analyzed the risks of individual permissions and collaborative permissions [41]. There are also some researchers extract permissions as [27] well as some other features [25] and use machine learning to detect malapps. Pemissions are the most commonly used and effective static feature in Android malicious apps detection. Although there are malicious behaviors in the apps code, these API calls still require permissions. So in Android malicious detection, permissions are more popular and effective than other features.

### 5.5 Performance of detection

In this section, we evaluate the performance of our method with 6 machine learning classifiers and 2 validation methods on different datasets.

The experimental results of Percentage Split and 10-fold cross validation are shown in Table 7.

**Table 7:** Calculation results for two models—Percentage Split and 10-fold cross validation.

Model	Classifier	TPR	FPR	Precision	Recall	F-M	ACC	Time
**10-fold cross validation**	BN	0.913	0.085	0.917	0.913	0.913	91.27%	0.04s
	NB	0.911	0.088	0.912	0.911	0.911	91.09%	0.01s
	**J48**	**0.988**	**0.012**	**0.988**	**0.988**	**0.988**	**98.70%**	**0.02s**
	RF	0.914	0.083	0.918	0.914	0.914	91.44%	0.26s
	RT	0.555	0.47	0.695	0.555	0.445	55.54%	0.01s
	K-NN	0.908	0.091	0.909	0.908	0.908	81.7%	0.01s
Percentage Split	BN	0.918	0.079	0.922	0.918	0.918	91.00%	0.05s
	NB	0.905	0.093	0.908	0.905	0.905	90.48%	0.07s
	J48	0.946	0.051	0.95	0.946	0.946	94.60%	0.02s
	RF	0.918	0.079	0.922	0.918	0.918	91.77%	0.21s
	RT	0.918	0.079	0.922	0.918	0.918	91.71%	0.02s
	K-NN	0.897	0.103	0.898	0.897	0.897	79.5%	0.02s

It can be seen from Table 7 that in addition to the Random Tree classifier, the results (TPR, FPR, Precision, Recall, F-Measure, Accuracy) of 10-fold cross validation are superior to those of Percentage Split. Therefore, 10-fold cross validation is used in the Android malware detection system for model selection.

It can be seen from Table 7 that comparing the experimental results of each machine learning classifier, the performance of J48 in the case of 10-fold cross validation (TPR, FPR, Precision, Recall, F-Measure(F-M), Accuracy) are better than other machine learning classifiers. Among them, TPR reached 98.8%, precision reached 98.8%, and Accuracy was 98.07%. In terms of speed, the algorithm is also efficient, as it took only 0.02s to train the model.

**Fig 5:**
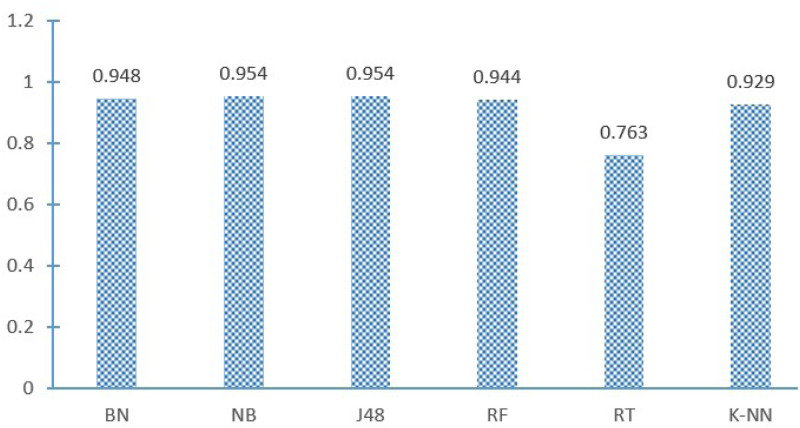
AUC of different classifiers.

The AUC of six classifiers (NB,BN,RT,J48,RF and K-NN) under 10-fold Cross Validation is shown in Fig 5.

It can be seen from the Fig 5, the AUC of all other classifiers except RT are higher than 0.9. Where NB and J48 can reach 0.954. Therefore, under 10-fold Cross Validation, the AUC of J48 is 0.954, indicating that the classification performance of the J48 is good.

The proposed detection method is verified with different datasets. The experimental results are shown in Table 8. With the increase of the scale of the datasets, the accuracy of the proposed approach is improved. For datasets with different sizes, the detection accuracy is all higher than 98%. For dataset3 the detection accuracy achieve up to 99.5%. The training and testing time only 0.05s. Therefore, the proposed approach is beneficial for large-scale datasets.

**Table 8:** Experimental results for different dataset.

Dataset	Number of samples	TPR	FPR	Precision	Recall	F-M	ACC	Time(s)
Dataset1	1145	0.988	0.012	0.988	0.988	0.988	98.70%	0.02
Dataset2	3994	0.991	0.021	0.991	0.991	0.991	99.10%	0.03
**Dataset3**	**10131**	**0.995**	**0.021**	**0.995**	**0.995**	**0.995**	**99.50**%****	**0.05**

The method based on TF-IDF and machine learning can quickly and efficiently detect malwares. Therefore, the proposed approach is suitable for classification of malicious apps.

### 5.6 Detection of malware families

In this section, the proposed approach is used to classify malicious families. The used dataset is dataset4. All these malicious families are known, such as FakeInstaller, Opfake, GingerMaster, DroidKungFu, BaseBridge, Iconosys, etc.

We implemented a classification experiment on the top 20 malicious families. The classification results are shown in Table 9.

**Table 9:** Experimental results for different families.

Malware Family	Number of samples	FPR	Precision	AUC
FakeInstaller	925	0	100%	1
DroidKungFu	662	0	100%	1
Plankton	553	0	100%	1
Opfake	612	0	100%	1
GinMaster	338	0	100%	1
BaseBridge	315	0	100%	1
Iconosys	135	0	100%	1
Kmin	96	0	100%	1
FakeDoc	132	0	100%	1
Adrd	82	0.1%	96.4%	0.904
Geinimi	84	0.2%	88.9%	0.823
DroidDream	78	0	100%	1
Glodream	68	0	100%	0.98
MobileTx	69	0.1%	94.5%	0.938
ExploitLinuxLotoor	68	0	100%	1
FakeRun	61	0	100%	1
SendPay	59	0	100%	1
Gappusin	46	0	100%	1
Imlog	43	0	100%	1
SMSreg	40	0	100%	1
Weighted Avg.	4466	0	99.6%	0.993

It can be seen from Table 9. For the top 20 malicious families, the detection accuracy of the proposed method is higher than 88.9%. The detection precision of the other 17 families has reached 100%, except for the three families of Adrd, Geinimi and MobileTx. According to Table 9, when the proposed method classifies malicious families, the AUC of 16 families are 1 and the AUC of 3 families are greater than 0.9. Meanwhile, the weighted average value of the AUC reached 0.993. Therefore, the proposed method also has a good effect on the classification of malicious families.

Endroid [5], Droidsieve [18] and our method use the same datasets to detect malware families. Endroid [5] dynamically extract features, whereas our method and Droidsieve [18] do it statically. The accuracy of the proposed approach achieves up to 99.6% is higher than Droidsieve (98.2%) and Endroid (94.5%). At the same time, the proposed approach only uses permission feature, while Droidsieve and Endroid use multiple features. They use multiple features for detection, which complicates the detection system. In addition, the proposed method also has a good detection accuracy for malware families.

### 5.7 Detection of unknown/ new apps

As described before, we have collected 892 real-world datasets (dataset5). The proposed method is verified by using the dataset5. The detection results of unknown/ new apps are shown in Table 10. According to Table 10, the detection precision can reach 93.3% and ACC can reach 92.71%. It shows that our proposed method also has a high detection accuracy for unknown and new apps.

**Table 10:** Experimental results for unknown dataset.

Dataset	TPR	FPR	Precision	Recall	F-M	ACC
Dataset5	0.927	0.067	0.933	0.927	0.927	92.71%

### 5.8 Comparison with other approaches

The proposed approach also is compared with other state-of-the-art malicious detection methods that only use permissions features. SIGPID [27] is an approach that applies permission ranking. We reimplemented their approach for comparison. Because the dataset used is different, the results are different from theirs.

The comparison results are shown in Table 11. SIGPID using only 22 significant permissions to classify different families of Apks. Compared with SIGPID, the F-M of our method is 99.8%, and the SIGPID is 98.7%. SIGPID takes 14 times as long to learn and test data as our method. Our method has higher F-M and less training and learning time. Meanwhile, if we only use the 24 dangers permissions (24 Dan-Per) of Google stated for detection. Its detection accuracy rate is 83.7%, far lower than the proposed method.

**Table 11:** Comparison with other state-of-the-art detection approaches.

Methods	Features	Samples(Mal./Ben.)	Classifier	F-M	ACC	Time(s)
SIGPID	Permission	Dataset3	J48	98.7%	98.7%	0.7
24 Dan-Per	Permission	Dataset3	J48	83.7%	83.7%	0.9
**Our method**	**Permission**	**Dataset3**	**J48**	**99.5%**	**99.5%**	**0.05**

### 5.9 Open issues for future work

Despite the effectiveness of the proposed method, there are several open issues.

Firstly, permission has a good detection effect in static detection, but other features such as API, Intent, etc. also have a good effect in Android security detection [59]. DroidSieve [18] and Drebin [57] use multiple features (including permission, API calls, code structure and invoked components) to detect malwares. The proposed method for android malware detection is based only on permissions. In future work, other features (such as API calls, intent, Network address, etc.) should be considered. Meanwhile, our model based on apps’ permissions is relatively small. According to our study, nearly half of the apps also used customized permissions models. In the following studies, we will consider custom permissions.

Secondly, our method uses NB, BN, RT, J48, RF and *K*-NN six classifiers to analyze the malicious detection effect of Android apps. Some ensemble learning methods [60, 61] and deep learning methods [62] have a good effect on the classification. In the following research, we will consider these learning methods.

Thirdly, identifier renaming, string encryption, Java reflection, packing and control flow obfuscation technology are widely used in Android apps [18]. The obfuscation of apps affects the detection effect. In the following work, we will consider the detection techniques for different obfuscation methods.

Fourthly, Static analysis does not take care the environment and state of the app at runtime. In some malicious Apks the permissions declared are not compatible with the permissions used. The hybrid approach [63, 64] combines the advantages of static analysis and dynamic analysis. It can be seen as the most comprehensive analysis because it analyzes both Android application installation files and behaviors of the app at runtime. In future studies, we will consider the combined use of dynamic and static detection methods.

## 6 Conclusions

In this paper, we discuss the importance of android system permission in android app’ security. Only use dangerous permissions or the number of used permissions can’t accurately distinguish whether it is a malicious app or a benign app. We used text mining approach (TF-IDF) to extract the system permission feature in the android app’s manifest.xml. The extracted features(the sensitive values permissions) are trained and classified by the machine learning algorithm. It is found that the accuracy of the proposed model is higher under the 10-fold cross-validation method and the J48 classifier. The TPR of the proposed method reaches 2.1%; the precision reaches 99.5%, and the ACC reaches 99.5%. So the proposed method has a high accuracy. Meanwhile, the training model only needs 0.05s with high efficiency. According to experiments, the proposed method is also applicable to different sizes of datasets. The detection accuracies of different sizes of datasets are all higher than 98%. The proposed method is also suitable for large-scale malwares detection. For 20 common malware families, the detection accuracy of the proposed method are 99.6%. The malware detection accuracy is better than some state-of-the-art malicious detection methods. Meanwhile, the method is effective for the unknown and new apps’ detection, and the accuracy of detection reaches 92.71%. Therefore, the proposed method is simple, feasible and efficient for android apps security detection.
